# A case-matched study of imatinib mesylate between different formulations on plasma trough concentration, adverse events, quality of life and outcomes in gastrointestinal stromal tumor patients

**DOI:** 10.1371/journal.pone.0303290

**Published:** 2024-05-14

**Authors:** Li Zeng, Xiang Cheng, Juan Li, Jun Zhang, Xingye Wu

**Affiliations:** 1 Department of Combination of Chinese and Western Medicine, The First Affiliated Hospital of Chongqing Medical University, Chongqing, China; 2 Clinical Center for Tumor Therapy, The Second Affiliated Hospital of Chongqing Medical University, Chongqing, China; 3 Department of Pharmacy, The First Affiliated Hospital of Chongqing Medical University, Chongqing, China; 4 Department of Gastrointestinal Surgery, The First Affiliated Hospital of Chongqing Medical University, Chongqing, China; Istanbul University-Cerrahpaşa, Cerrahpaşa Faculty of Medicine, TURKEY

## Abstract

Genike, the imatinib (IM)-alpha form is widely used in the treatment of gastrointestinal stromal tumor (GIST) patients in China. We wanted to investigate whether there are differences in IM plasma concentrations, adverse events, health-related quality of life (QOL) and outcomes between patients treated with Genike and Glivec. Thirty included GIST patients receiving IM treatment were matched to either Genike or Glivec according to gastrectomy, body weight, body surface area and sex. There was no statistically significant difference in IM trough plasma levels between the two groups. There were no significant differences in very common adverse events of IM between the Genike and Glivec groups. IM was well tolerated, although it was associated with a significant change in cognitive function (*P* < 0.001), fatigue (*P* = 0.015), pain (*P* = 0.015), nausea/vomiting (*P* = 0.029), insomnia (*P* = 0.019), diarrhea (*P* = 0.003) and financial difficulties (*P* < 0.001). Physical functioning, financial burden and insomnia were significantly different between the two groups (*P* = 0.026). Until Aug. 2022, there was no significant difference in time to imatinib treatment failure (TTF) between the two groups. In conclusion, there was no difference in IM plasma concentration and adverse events between Genike and Glivec. Both Genike and Glivec could partially decrease the QOL of GIST patients. Physical functioning was worse in Genike group than in Glivec group, while the economic burden and symptoms of insomnia in Glivec patients were worse. There was no significant difference in TTF between the two groups.

## Introduction

Gastrointestinal stromal tumors (GISTs) are the most common mesenchymal tumor of the digestive tract and commonly occur in the stomach and small intestine. The molecular characteristics of GISTs include KIT or platelet-derived growth factor receptor alpha (PDGFRA) driver mutations, which are detectable in > 90% of cases [1,2]. Major advances have been made in the treatment of this disease due to the advent of tyrosine kinase inhibitors (TKIs), especially imatinib, which was the first TKI approved for the treatment of advanced GIST patients. The success of these agents in advanced disease prompted interest in their use in the preoperative setting as induction therapy for patients with unresectable or borderline resectable tumors and as adjuvant treatment for patients at high risk of recurrence after complete resection of a primary GIST tumor [3–7].

The overall survival of advanced GIST patients increased from 19 months, for those diagnosed before 2002 to approximately 55 months for those diagnosed after 2002. As treatment with imatinib may last for several years, drug tolerance is especially important. Even mild adverse events play a role in drug discontinuation [8]. On the other hand, it is known that drug discontinuation is one of the major problems in achieving complete response [9]. At the same time, the major concern for most patients has shifted from survival to a better quality of life (QOL) [10]. Our previous study found that there was a positive correlation between imatinib dosage and plasma concentrations [7]. Many scholars believe that TKI adverse events are related to imatinib dose. In addition, there is a clear correlation between imatinib plasma concentrations and the efficacy, adverse reactions and QOL of patients with GIST.

In China, previous studies have demonstrated the bioequivalence of Genike and Glivec in healthy volunteers [11]. A few years ago, Genike, IM-alpha form, was used to treat leukemia patients and had achieved similar efficacy and safety with Glivec for chronic myeloid leukemia in chronic phase [12]. Recently, Genike was widely used in the treatment of GIST patients. Still, no study has reported comparison results between Genike and Glivec in GIST patients. This study was carried out with the aim of comparing imatinib plasma concentrations, adverse events, QOL and time to imatinib treatment failure (TTF) in patients treated with Genike or Glivec.

## Materials and methods

### Patient inclusion

Patients with GIST receiving treatment with imatinib (produced in China (Genike) or Switzerland (Glivec)) from April 2017 to August 2018 were included in the study and their follow-ups were performed by the same surgeon. Patients were excluded if they had any serious comorbidity or if oral administration was restricted because of significant gastrointestinal bleeding or obstruction. Patients treated with drugs known to induce or inhibit CYP3A4 or P-glycoprotein (P-gp) and inhibitor of human organic cation transporter 1 (hOCT-1) were also excluded from the study if no alternative medication was available or if the patient was unwilling to change medications. Inclusion criteria were as follows: (1) pathologically confirmed GIST; (2) at least one trough imatinib plasma trough level (C_min_) could be obtained after a minimal 3 months of treatment (according to Eechoute et al. [13]); and (3) good compliance (take imatinib regularly). In our previous study [7], it was found that gastrectomy, body weight, body surface area and sex were significantly correlated with imatinib trough plasma concentration in Chinese patients. Therefore, the patients included were matched between the Genike and Glivec groups according to the above four factors. This study was conducted in accordance with the World Medical Association Declaration of Helsinki and approved by the Medical Ethical Committee of the First Affiliated Hospital of Chongqing Medical University (ethical approval code: 2019–162). All persons gave their informed consent prior to their inclusion in the study. Informed consent was obtained from all subjects.

### Treatment and assessment

The paired patients had received once-daily 400 mg imatinib produced in Genike or Glivec. A 3 ml blood sample was collected just before an imatinib dose (generally 24 ± 3 h following the previous dose) into a heparinized vial. The protocol for the determination of imatinib plasma concentrations was established according to the method described in our previous study [7]. The very common adverse events of imatinib (incidence≥10%) were assessed through an interview before medication and at least 3 months after medication. Adverse events were graded according to the National Cancer Institute Common Terminology Criteria for Adverse Events, version 4.03. Health-related QoL was assessed by the European Organization for Research and Treatment Quality of Life Questionnaire C30 (EORTC QLQ-C30), version 3.0 [14], which is a cancer-specific 30-item questionnaire that has been widely used in clinical trials. The EORTC QLQ-C30 questionnaire comprises a global health status/QOL scale, five functional scales (physical, role, emotional, cognitive, and social), three symptom scales (fatigue, nausea/vomiting, pain), and six single items (dyspnea, insomnia, appetite loss, constipation, diarrhea, and financial difficulties). All domains and scales were converted to scores ranging from 0 to 100 according to the scoring manual. A higher score for a functional or global QOL scale represents a relatively higher/healthier level of functioning or global QOL, whereas a higher score for a symptom/item scale represents a more severe symptom/problem. QOL was assessed along with adverse events. To avoid potential bias caused by the intervention of the attending surgeon, all patients were asked to fill out a QOL questionnaire in the waiting area before meeting the surgeon. TTF is an evaluation indicator that combines effectiveness and toxicity. It was determined by the period from the start of imatinib treatment to treatment discontinuation or termination, including any reason such as disease progression, death, withdrawal due to adverse events, or use of a new treatment.

### Statistics

Statistical analysis was performed using SPSS 22.0 software (Chicago, USA). A nonparametric test was used to compare the data of adverse events between the two groups. Imatinib plasma trough levels and baseline QOL parameters were compared using the paired t test between the two groups. Analysis of variance (ANOVA) with two-way repeated measures was used to compare the evolution of QOL parameters with time between the two groups. A two-sided *P* value < 0.05 was considered statistically significant.

## Results

### Patient characteristics

A total of 30 included patients were included in the analysis. The clinical characteristics of these patients are listed in Table 1. The two groups were similar in sex and surgical procedures. For each group, 7 were women (46.67%), and 8 were men (53.33%). Three patients with gastrectomy, 8 patients with nongastrectomy and 4 patients without surgery were included in each group. The mean body weight was 60.60 ± 9.41 kg in the Genike group and 60.20 ± 10.22 kg in the Glivec group, while the mean body surface area was 1.60 ± 0.16 m^2^ and 1.60 ± 0.17 m^2^ for each group, respectively. There were no significant differences in the above 4 variables between the Genike and Glivec groups.

**Table 1:** Comparison of predefined variables between Genike and Glivec groups.

Characteristic	Group		*P* Value
	Genike	Glivec	
Gender, n (%)			
Female	7 (46.67)	7 (46.67)	1
Male	8 (53.33)	8 (53.33)	1
Body weight (kg)	60.60±9.41	60.20±10.22	0.7078
Body surface area (m^2^)	1.60±0.16	1.60±0.17	0.9800
Surgical procedures, n (%)			
Gastrectomy	3 (20.00)	3 (20.00)	1
Non-gastrectomy	8 (53.33)	8 (53.33)	1
Without surgery	4 (26.67)	4 (26.67)	1

There were no statistically significant differences in all predefined variables (*P* ≥ 0.05).

### Plasma trough levels

**Fig 1:**
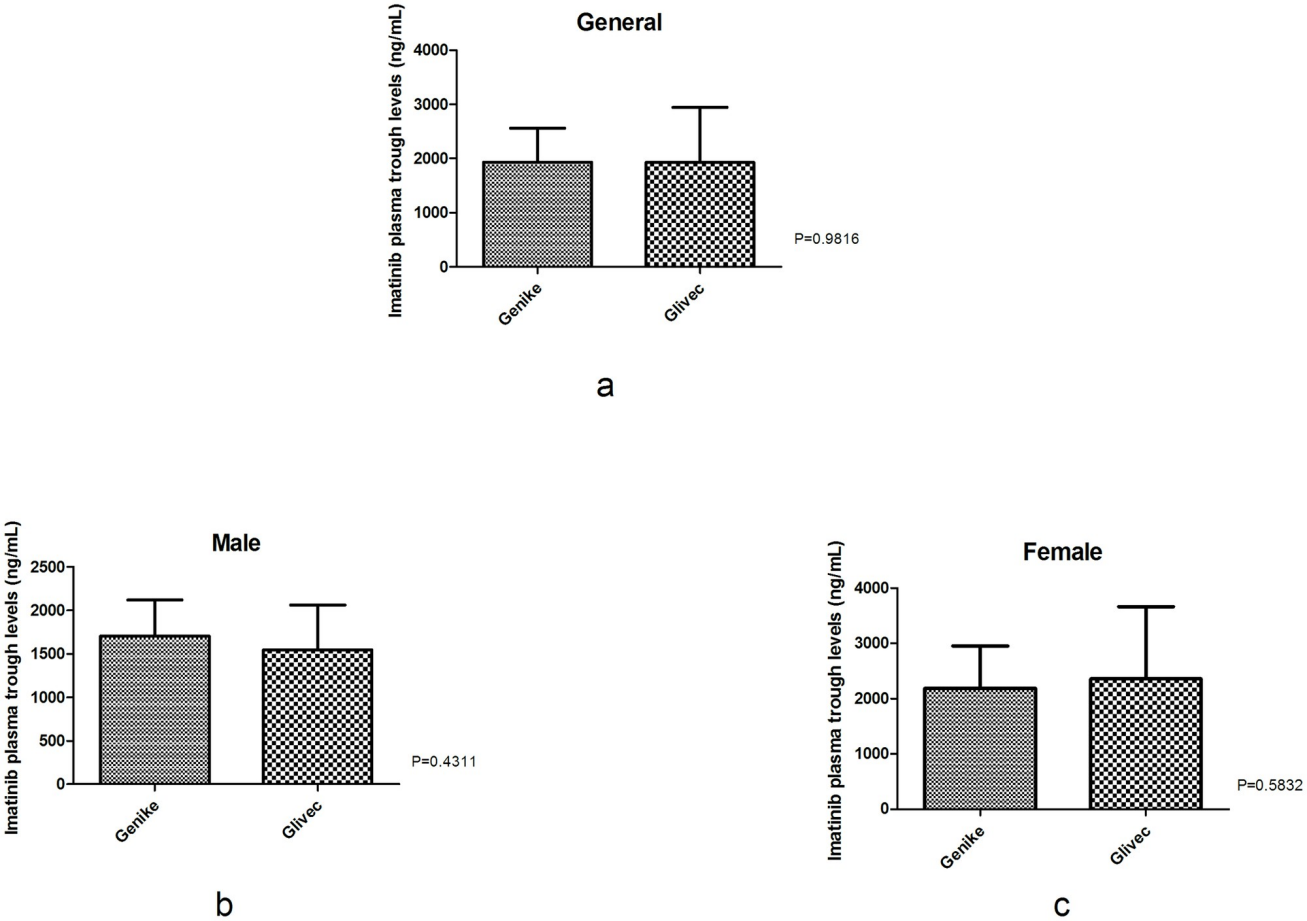
Comparison of imatinib trough plasma levels (Cmin) between the Genike (IM-C) and Glivec (IM-S) groups. There was no statistically significant difference between the two groups for (a) total, (b) male, or (c) female patients. (medication: Drug varieties of intervention; time: Before or after drug intervention; interaction: Interaction between medication and time).

The imatinib trough plasma levels of all patients in each group were 1930.72 ± 631.37 and 1926.75 ± 1018.86 ng/mL, respectively. The imatinib trough plasma levels of male patients in each group were 1704.23 ± 415.78 and 1547.82 ± 511.63 ng/mL, respectively, while the imatinib trough plasma levels of female patients in each group were 2189.55 ± 762.81 and 2359.81 ± 1306.35 ng/mL, respectively. There was no statistically significant difference between the two groups for total, male, or female patients (*P* = 0.982, 0.431, 0.583, respectively, Fig 1a–1c).

### Adverse events

Seventeen kinds of very common adverse events of imatinib were assessed in the two groups: periorbital edema, facial edema, limb edema, headache, abdominal pain, myalgia, ostealgia, nausea, diarrhea, vomiting, dyspepsia, rash/eczema/dermatitis, fatigue, weight gain, neutropenia, thrombocytopenia, and anemia. Table 2 lists the adverse events that occurred in patients in the two groups, and no grade 4–5 adverse events occurred. There were no significant differences in any adverse events between the Genike and Glivec groups (*P* ≥ 0.05).

**Table 2:** Adverse events between Genike and Glivec groups.

Adverse events	Group	Grade, n			*Z* Value
		1	2	3	*P* Value
Periorbital edema	Genike	13	1	1	Z = 0
	Glivec	13	1	1	*P* = 1.000
Facial edema	Genike	14	1	0	Z = -1.000
	Glivec	15	0	0	*P* = 0.317
Limb edema	Genike	15	0	0	Z = -1.439
	Glivec	13	2	0	*P* = 0.150
Headach/ Abdominal pain/ myalgia	Genike	15	0	0	Z = 0
	Glivec	15	0	0	*P* = 1.000
Ostealgia	Genike	15	0	0	Z = 0
	Glivec	15	0	0	*P* = 1.000
Nausea	Genike	14	1	0	Z = -1.000
	Glivec	15	0	0	*P* = 0.317
Diarrhea	Genike	14	1	0	Z = -1.056
	Glivec	12	3	0	*P* = 0.291
Vomiting/ Dyspepsia	Genike	15	0	0	Z = 0
	Glivec	15	0	0	*P* = 1.000
Rash/Eczema/Dermatitis	Genike	9	5	1	Z = -0.073
	Glivec	10	2	3	*P* = 0.942
Fatigue	Genike	14	1	0	Z = -1.000
	Glivec	15	0	0	*P* = 0.317
Weight gain	Genike	14	1	0	Z = -1.000
	Glivec	15	0	0	*P* = 0.317
Neutropenia	Genike	10	0	0	Z = 0
	Glivec	10	0	0	*P* = 1.000
Thrombocytopenia	Genike	10	0	0	Z = 0
	Glivec	10	0	0	*P* = 1.000
Anemia	Genike	10	0	0	Z = -1.000
	Glivec	9	1	0	*P* = 0.317

There were no statistically significant differences in all adverse events (*P* ≥ 0.05).

### QOL analysis

There were no significant differences in all scores of QOL parameters at baseline between the two groups (*P* ≥ 0.05, Table 3).

**Table 3:** QOL parameters at baseline.

EORTC QLQ-C30	Genike		Glivec		*P* Value
	Mean	SD	Mean	SD	
Global health status/QOL	26.67	12.28	32.22	12.94	0.238
Functioning					
Physical	90.00	14.80	98.22	5.33	0.08
Role	100	0	100	0	1
Emotional	97.78	5.86	100	0	0.164
Cognitive	97.78	5.86	98.89	4.30	0.582
Social	98.89	4.30	100	0	0334
Symptoms					
Fatigue	1.11	4.30	1.47	3.87	0.824
Nausea/vomiting	0	0	0	0	1
Pain	1.11	4.30	2.22	5.86	0.582
Dyspnoea	0	0	4.44	11.73	0.164
Insomnia	6.67	25.82	0	0	0.334
Appetite loss	0	0	0	0	1
Constipation	2.22	8.61	2.22	8.61	1
Diarrhoea	0	0	2.22	8.61	0.334
Financial difficulties	0	0	2.22	8.61	0.334

There were no statistically significant differences in all scores (*P* ≥ 0.05).

**Fig 2:**
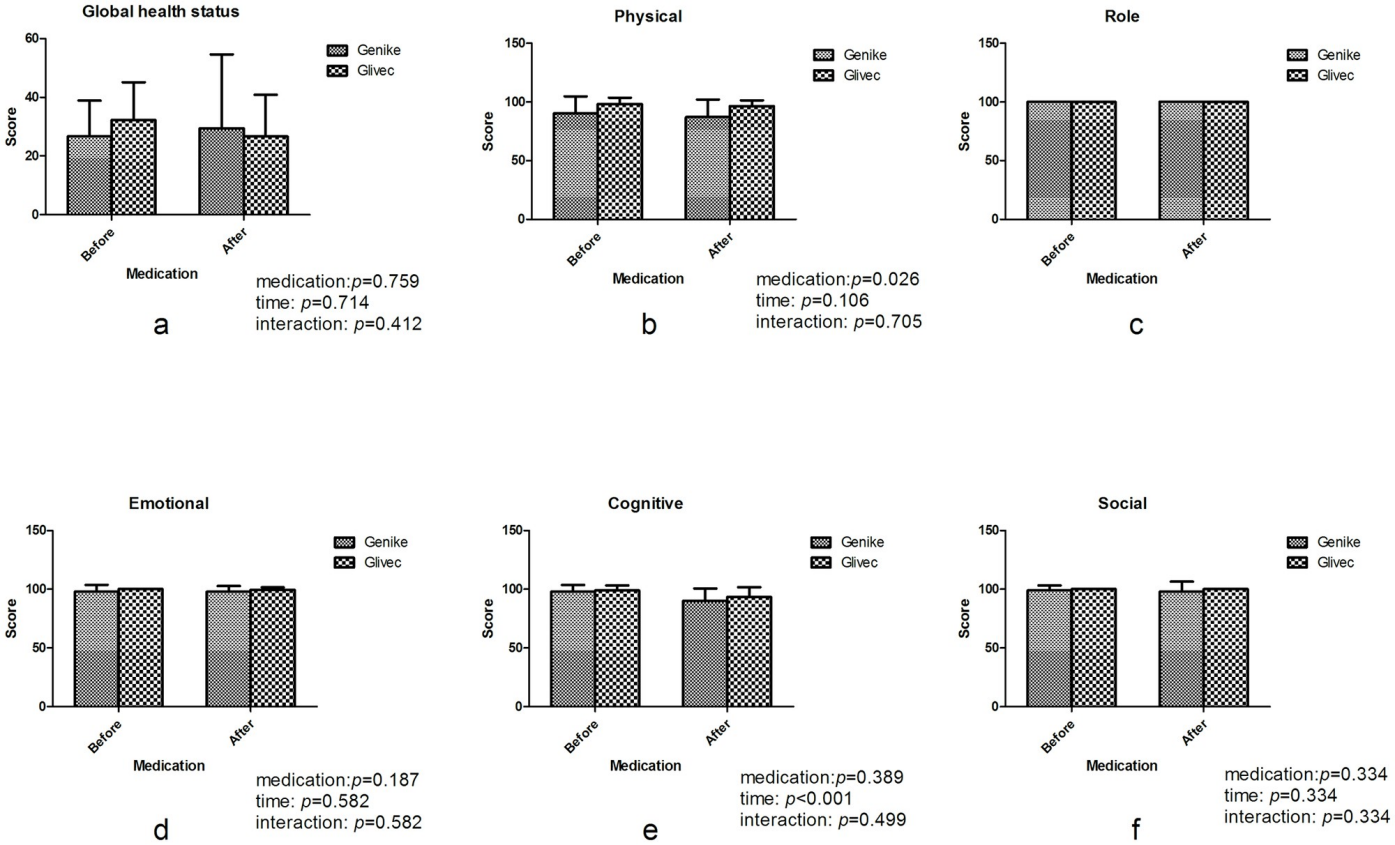
Comparison of the global health status and functional scores in patients with GIST between the Genike (IM-C) and Glivec (IM-S) groups. There were no statistically significant differences after medication in (a) global health status and (c) role, (d) emotional and (f) social functioning, while (b) physical functioning was worse in the Genike group (IM-C) than in the Glivec group (IM-S) (*P* = 0.026), and lower scores of (e) cognitive functioning were gained in both groups after medication (*P* < 0.001). (medication: Drug varieties of intervention; time: Before or after drug intervention; interaction: Interaction between medication and time).

In global health status and functional scales such as role, emotional and social functioning, there were no statistically significant differences after medication regardless of different medications, times or interactions (Fig 2a–2f). For physical functioning analysis, there was a slight reduction in both groups after medication, but there was no statistical significance (*P* = 0.106, Fig 2b), while physical functioning was worse in the Genike group than in the Glivec group (*P* = 0.026, Fig 2b). With regard to cognitive functioning analysis, there were no statistically significant differences between the two groups (*P* = 0.389, Fig 2e), while lower scores were obtained by both groups after medication (*P* < 0.001, Fig 2e).

**Fig 3:**
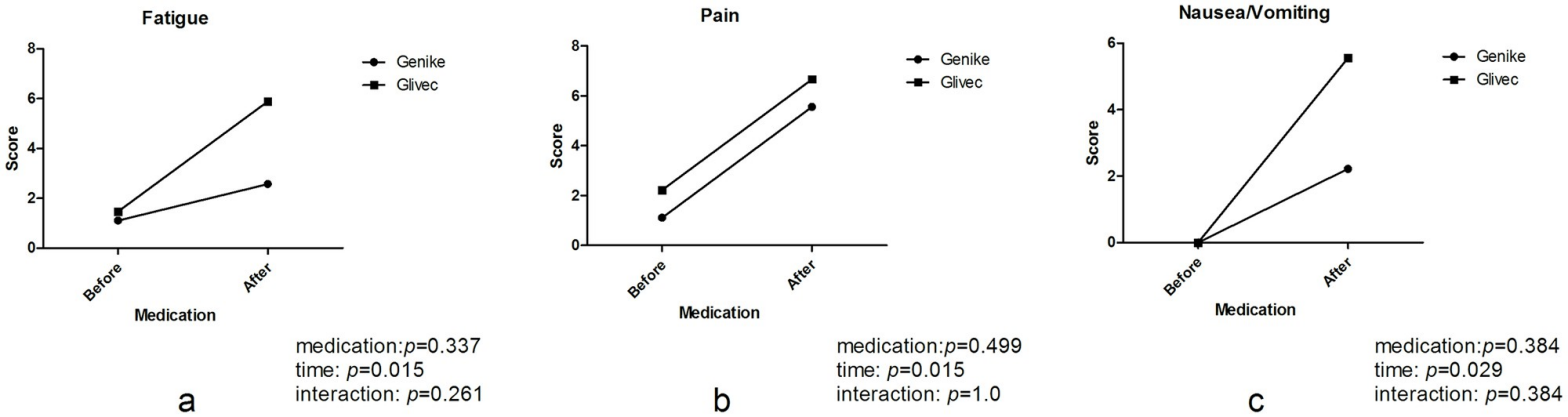
Comparison of the symptom scores in patients with GIST between the Genike (IM-C) and Glivec (IM-S) groups. (a) Fatigue, (b) pain, and (c) nausea/vomiting were more prevalent in both groups after medication (*P* = 0.015, 0.029, 0.015, respectively). (medication: Drug varieties of intervention; time: Before or after drug intervention; interaction: Interaction between medication and time).

**Fig 4:**
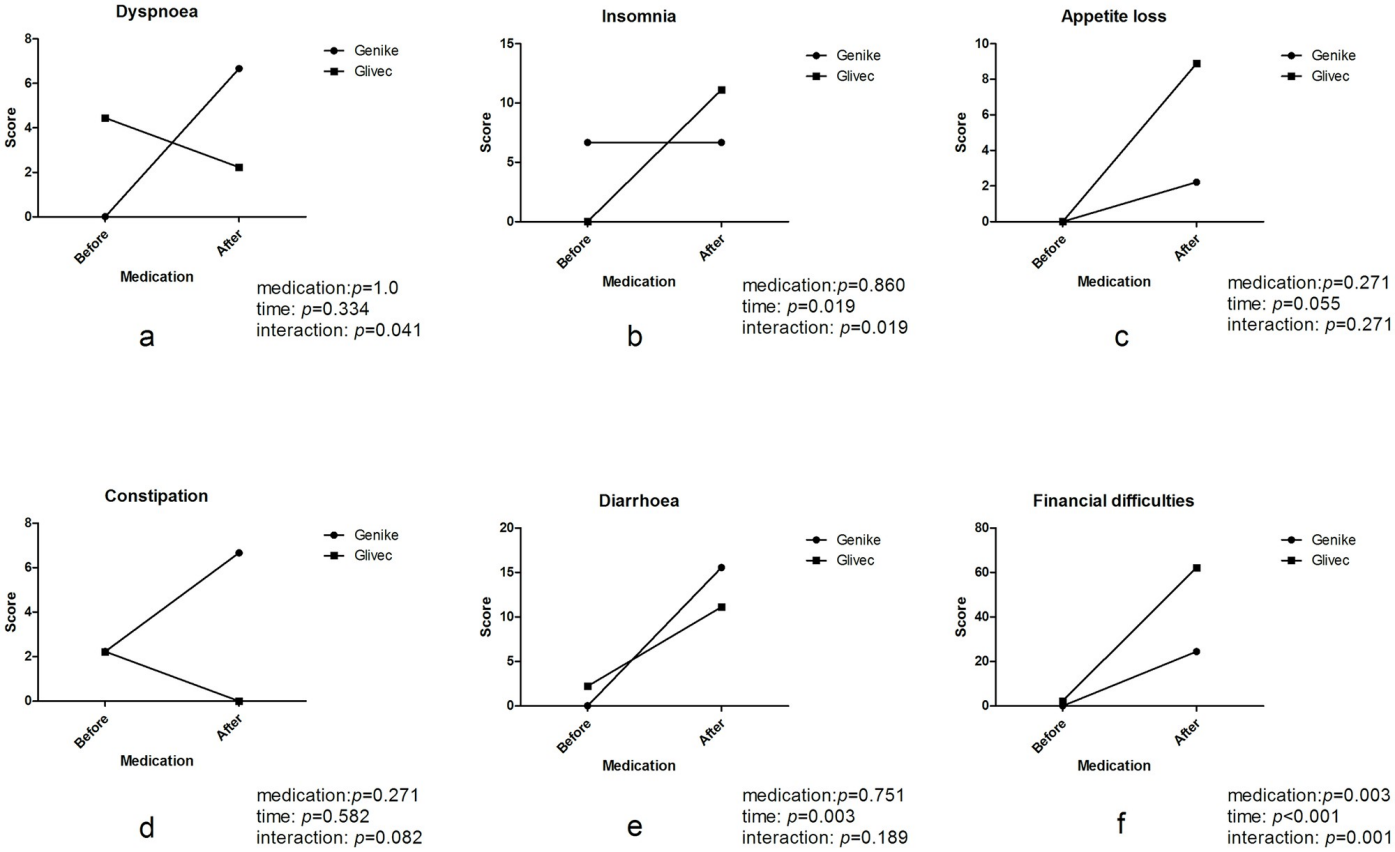
Comparison of the single item scores in patients with GIST between the Genike (IM-C) and Glivec (IM-S) groups. There were no statistically significant differences in (a) appetite loss, (c) constipation and (d) dyspnea between the two groups, and (e) diarrhea was more prevalent in both groups after medication (*P* = 0.003), while (b) insomnia was more prevalent only in the Glivec (IM-S) group after medication (*P* = 0.019). With regard to the analysis of (f) financial difficulties, they were more prevalent in both groups after medication (*P* < 0.001), especially in the Glivec group (IM-S) compared with the Genike group (IM-C) (*P* = 0.003). (medication: Drug varieties of intervention; time: Before or after drug intervention; interaction: Interaction between medication and time).

In the symptom/item scales, fatigue, pain, nausea/vomiting, and diarrhea were more prevalent in both groups after medication (*P* = 0.015, 0.029, 0.015, 0.003, respectively, Figs 3a–3c and 4e), while insomnia was more prevalent only in the Glivec group after medication (*P* = 0.019, Fig 4b). There were no statistically significant differences in appetite loss and constipation between the two groups, regardless of different medications, times or interactions (Fig 4c and 4d). With regard to the analysis of dyspnea, there was a significant interaction between the effects of medication and time (*P* = 0.041, Fig 4a), but there were no statistically significant differences regardless of medication or time. With regard to the analysis of financial difficulties, there was a significant interaction between the effects of medication and time (*P* = 0.001, Fig 4f), and they were more prevalent in both groups after medication (*P* < 0.001, Fig 4f), especially in the Glivec group compared with the Genike group (*P* = 0.003, Fig 4f).

### Clinical outcomes

Until Aug. 2022, 2 patients were lost to follow-up, 13 pairs of patients performed final analysis. In the Genike group, 1 patient had disease recurrence, and 1 patient died due to disease progression. None of the patients discontinued treatment due to adverse drug reactions. There was no significant difference in TTF between the two groups (*P* = 0.669).

## Discussion

Effectiveness, safety and economy are important factors in drug evaluation. Blood drug concentration is one of the important indicators of bioequivalence. This study was carried out with the aim of comparing the imatinib plasma concentrations, adverse events, QOL and TTF in patients treated with Genike or Glivec. The main finding of this study was that there was no statistically significant difference in imatinib trough plasma levels and TTF between the two groups. There were no significant differences in very common adverse events of imatinib between Genike and Glivec. Cognitive functioning, financial difficulties, fatigue, pain, nausea/vomiting, and diarrhea were more prevalent in both groups after medication. Physical functioning was worse in the Genike group than in the Glivec group, while the economic burden and symptoms of insomnia in Glivec patients were worse.

Population pharmacokinetic studies have suggested that the plasma concentration of imatinib may be influenced by white blood cell, granulocyte, hemoglobin, and α1-acid glycoprotein levels, age, body weight, body-surface area and chronic exposure to imatinib [15–19]. In our previous study, we investigated whether the factors affecting imatinib C_min_ in Chinese populations were the same as those in Western populations [7]. Gastrectomy, body weight, body surface area and sex may be factors affecting imatinib C_min_ in Chinese patients. In this study, 30 patients were matched between the Genike and Glivec groups according to the above four factors. The main finding of this study was that there was no statistically significant difference in IM trough plasma levels and TTF between the two groups. Genike is a capsule, but Glivec is a tablet. Imatinib was initially formulated and approved as a capsule, and new formulations of tablets have since been developed. Similar to the capsules, the dissolution profiles of the 100 mg and 400 mg tablets were similar to the profile of the 100 mg capsules. The bioequivalence of the 100 mg and 400 mg tablet formulations compared with the standard 100 mg capsule formulation was evaluated in 33 healthy volunteers [20]. Both the 100 mg and 400 mg tablet formulations of imatinib were bioequivalent to the standard 100 mg hard-gelatin capsule [20]. Gao et al. reported that the major pharmacokinetic parameters of Genike and Glivec were basically consistent and that the two drugs were bioequivalent in 24 healthy Chinese volunteers [11]. Our results suggest that imatinib blood concentration is not affected by dosage forms at the same time and dose.

The TKI imatinib is now the first-line therapy for GIST. Nearly all imatinib-treated patients experience at least one side effect of any grade of severity [4]. However, it has been well tolerated in clinical studies with adverse events typically being mild to moderate in healthy volunteers, and patients with chronic myeloid leukemia, GIST and other cancers, usually managed without permanent discontinuation of therapy [21]. In this research, the very common adverse events of imatinib were assessed, including periorbital edema, facial edema, limb edema, headache, abdominal pain, myalgia, ostealgia, nausea, diarrhea, vomiting, dyspepsia, rash/eczema/dermatitis, fatigue, weight gain, neutropenia, thrombocytopenia and anemia. Consistent with previous research findings, almost every patient had at least a grade 1 or 2 adverse event. No patient withdrew from this study because of adverse events. There were no significant differences in all adverse events between Genike and Glivec. A multicenter retrospective clinical study to evaluate the efficacy and safety of Genike and Glevic in newly diagnosed patients with chronic myeloid leukemia in chronic phase. They find that Genike was well tolerated, and there were no statistically significant differences in efficacy and safety between Genike and Glivec. This result consistent with our research in GIST [12]. Many scholars believe that TKI adverse events are related not only to imatinib dose but also to treatment duration, age, sex and cultural differences [22]. In our previous research, we found that gastrectomy, body weight, body surface area and sex may be factors affecting imatinib C_min_ in Chinese patients. In this study, the included patients were matched according to imatinib dose, treatment duration, and sex. This may be one of the reasons leading to no difference in adverse reactions between Genike and Glivec.

Over the past decade, GIST has been the soft tissue sarcoma with the most significant change in life expectancy due to the advent of targeted therapies. The major concern for most patients has shifted from survival to a better QOL. Complete evaluation of treatment benefits or disadvantages involves not only the assessment of efficacy and safety but also QOL. In the present study, we investigated the effects of Genike and Glivec being used in GIST treatment on QOL. Imatinib was well tolerated, although it was associated with a significant increase in cognitive function, fatigue, pain, nausea/vomiting, insomnia, diarrhea and financial difficulties. Comparison of QOL parameters between the two groups did not reveal significant differences except physical functioning and financial difficulties. Physical functioning was worse in the Genike group than in the Glivec group, while financial difficulties and insomnia symptoms were worse in the Glivec group. The very high cost of TKIs is a well-known problem. A study of 1541 patients with chronic myeloid leukemia showed that patients with higher copayments are more likely to discontinue or be nonadherent to TKIs. In the long term, the heavier economic burden may lead to more serious consequences [23]. In addition, there was a tendency without statistical significance that dyspnea and constipation were better in the Glivec group than in the Genike group.

In conclusion, we found no difference in imatinib plasma concentration, adverse events or TTF between the Genike and Glivec groups. Resumption of imatinib was well tolerated, but Genike or Glivec could decrease the QOL of GIST patients. Physical functioning was worse in the Genike group than in the Glivec group, while financial difficulties and insomnia symptoms were worse in the Glivec group. It is of great significance and value to verify the efficacy and safety of Genike, the imatinib-alpha form. Because it is cheaper than the original molecule, it is widely used in developing countries. As a practical limitation, the sample size of this study was small, so in future studies, the sample size should be increased. We hope to obtain more accurate and comprehensive results in future studies.

## Supporting information

S1 File (XLSX)
